# Involvement of Orotic Acid in Mitochondrial Activity of Ovarian Granulosa Cells and Oocyte Meiotic Maturation

**DOI:** 10.3390/ijms26104479

**Published:** 2025-05-08

**Authors:** Weronika Marynowicz, Aleksandra Tatarczuch, Zuzanna Flis, Edyta Molik, Anna Ptak

**Affiliations:** 1 Laboratory of Physiology and Toxicology of Reproduction, Institute of Zoology and Biomedical Research, Faculty of Biology, Jagiellonian University, Gronostajowa 9, 30-387 Krakow, Poland; weronika.marynowicz@doctoral.uj.edu.pl (W.M.); aleksandra.tatarczuch@doctoral.uj.edu.pl (A.T.); 2 Doctoral School of Exact and Natural Sciences, Jagiellonian University, Prof. St. Łojasiewicza St 11, 30-348 Krakow, Poland; 3 Department of Animal Nutrition and Biotechnology, and Fisheries, Faculty of Animal Science, University of Agriculture in Krakow, al. Mickiewicza 21, 31-120 Krakow, Poland; flis.zuzanna@gmail.com (Z.F.); edyta.molik@urk.edu.pl (E.M.)

**Keywords:** orotic acid, cell metabolism, in vitro maturation, human ovarian cells, mouse oocytes

## Abstract

Orotic acid (OA) is a natural component of milk and is found in many biological fluids such as human ovarian follicular fluid. However, its effect on ovarian cells is unknown. Some studies suggest that OA may alter lipid metabolism and energy production in cells. In the present study, we determine the effect of OA on mitochondrial function and lipid droplet content in the human granulosa cell line. The effect of OA on in vitro mouse oocyte maturation and mitochondrial activity was also investigated. We found that repeated exposure to OA (0.01–1000 µM) did not alter the viability of human epithelial (HOSEpiC) and granulosa (HGrC1) ovarian cells. HGrC1 cells treated with a high dose of OA (500 µM) showed a more aerobic and energetic phenotype than control cells, whereas this effect was not observed after treatment with lower doses (0.01 and 100 µM) of OA. In addition, OA at a high dose (500 µM) reduced lipid droplet (LD) content without altering glucose (GLUT1, GLUT4) and fatty acid transporter (SLC27A1) gene expression in HGrC1 cells. At the same time, OA at 100 µM did not disrupt mouse in vitro oocyte maturation, whereas OA at 500 µM inhibited this process by arresting oocytes at the germinal vesicle (GV) stage with a reduction in mitochondrial activity. Our results show that OA at high doses can disrupt female reproduction, but normal dietary orotate intake does not have a negative effect on ovarian function.

## 1. Introduction

Orotic acid (OA) is a natural component of milk and an intermediate in the pyrimidine biosynthetic pathway [1,2]. OA is found in many biological fluids, and the major dietary sources of this component in humans are bovine milk (~80 mg/L) and sheep milk (~300 mg/L) [3]. However, low levels of OA have been found in human milk (<10 mg/L) [4]. The positive effects of OA products claimed for humans are highlighted by promises made in the public domain, such as beneficial cardiovascular effects, energy supply, improvement in body composition and enhanced athletic performance [5,6]. Orotate therapy has previously been shown to improve energy supply and contractile function in normal and ischaemic rat hearts [7]. Moreover, studies have suggested that OA has a beneficial effect on glycogen levels in electrically stimulated myotubes and that OA may influence basic signalling cascades involved in both metabolic and inflammatory adaptation processes of skeletal muscle to exercise [6]. In addition, OA preserved pancreatic b-cell function and reduced cell death and may therefore be a future therapeutic strategy for the prevention and treatment of diabetes in the mouse model [8]. On the other hand, excessive dietary orotic acid caused severe hepatic steatosis and hepatomegaly with decreased serum lipoprotein export in rats [9]. Therefore, OA may disrupt lipid homeostasis by affecting both fatty acid and cholesterol synthesis. In turn, lipids are essential components of cells involved in metabolic, endocrine, and reproductive functions, such as ovarian cells. Therefore, with the growing interest in nutraceuticals such as OA, more research is urgently needed to confirm their safety for ovarian health. However, the effects of OA on the reproductive system have not been described.

The ovarian follicle, the basic reproductive unit of the ovary, is the key to female fertility and consists of oocytes and follicular somatic cells, including granulosa and theca cells. Granulosa cells provide the oocyte with essential nutrients, growth factors, and metabolic precursors [10]. The main metabolic pathways contributing to energy homeostasis are glycolysis and oxidative phosphorylation, which couple the breakdown of nutrients such as glucose, amino acids and fatty acids with the production of ATP. Within the ovarian follicle, lipid metabolites play an important role in cell signalling and metabolic processes. For example, fatty acids are the energy providers in oocytes [11], and cholesterol is the precursor of steroid hormones that are synthesised in granulosa and theca cells. On the other hand, excessive lipid accumulation can severely damage ovarian reproductive function by inducing ovarian oxidative stress and inflammation [12]. Therefore, OA may affect ovarian follicular function by regulating cellular metabolic processes.

Here, we investigated whether repeated exposure to OA affects the viability of human ovarian epithelial and granulosa cell lines. The effect of OA on mitochondrial energy production and lipid accumulation in human granulosa cells was also investigated. Finally, we investigated whether OA could affect the in vitro maturation and mitochondrial activity of mouse oocytes.

## 2. Results

### 2.1. Effects of Repeated Exposure of OA on the Viability of HOSEpiC and HGrC1 Cells

**Figure 1:**
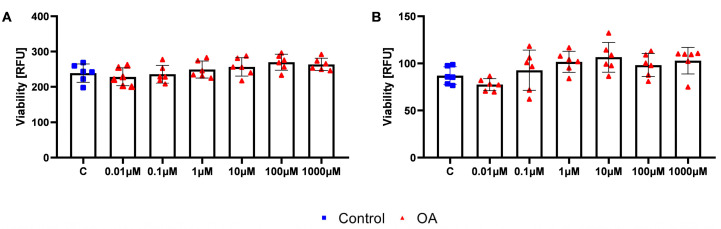
Viability of human ovarian epithelial (HOSEpiC) and granulosa cells (HGrC1) after repeated exposure to orotic acid (OA). Effect of orotic acid (0.01, 0.1, 1, 10, 100, and 1000 µM) on the viability of HOSEpiC (**A**) and HGrC1 (**B**) after repeated exposure. C, control (0.1% DMSO). One-way ANOVA followed by Tukey’s multiple comparison test was used to determine statistical significance. Each bar represents the mean ± SD; the dots represent biological replicates of the experiments.

First, we analysed the effect of OA on the viability of human follicular cells after repeated exposure. We exposed both epithelial and granulosa cells twice to OA at 0.01–1000 µM and measured cell viability after 48 h of exposure using the Presto blue assay. OA at all doses tested had no effect on the viability of either cell type compared to control cells after repeated exposure (Figure 1A,B).

### 2.2. Effects of Repeated Exposure of OA on the Metabolic Profile of HGrC1 Cells

**Figure 2:**
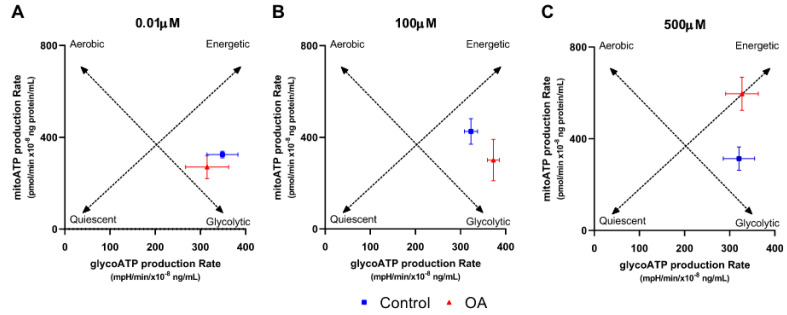
Energy map for human granulosa (HGrC1) cells cultured under different concentrations of orotic acid. The effect of orotic acid (OA) at (**A**) 0.01 µM, (**B**) 100 µm, and (**C**) 500 µM on extracellular acidification rates (ECAR, gylcoATP) versus oxygen consumption rates (OCR, mitoATP). Dotted lines represent the aerobic–glycolytic axis and the quiescent-energetic axis. C, control (0.1% DMSO). Data are expressed as the mean ± SD of three independent experiments.

The most important functional cells in the ovary are the granulosa cells. As previous data have suggested that OA may affect the metabolic activity of cells [5], we analysed the effect of repeated exposure to 0.01 µM, 100 µM, and 500 µM OA on the metabolic profile of human granulosa HGrC1 cells. We observed differences in the energy profile only after treatment with 500 µM OA (Figure 2A–C). Cells treated with a high dose of OA (500 µM) show a more aerobic and energetic phenotype than control cells (Figure 2C).

**Figure 3:**
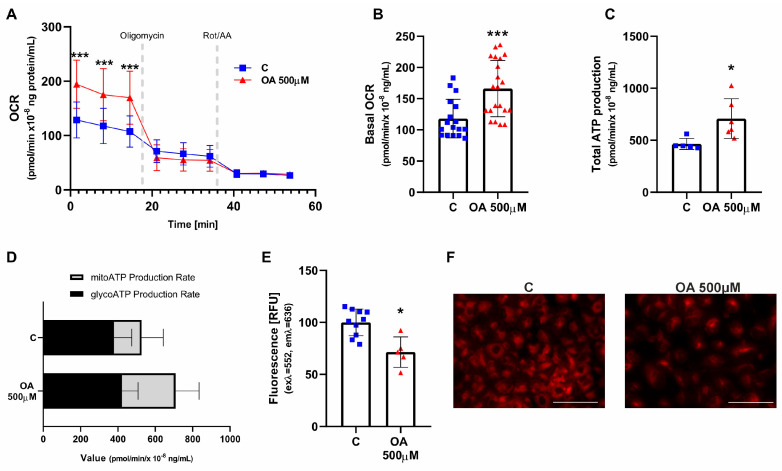
ATP production profile and lipid content after orotic acid (OA) exposure in HGrC1 cells. The effect of 24 h treatment with OA (500 µM) on OCR, kinetic data plot of the XF ATP rate assay (**A**), basal oxygen consumption rate (OCR) (**B**), total ATP production rate (**C**), dominant process for ATP production (**D**), and lipid droplet content (**E**). Representative images of Nile red staining (**F**). C, control (0.1% DMSO). Student’s *t*-test was used to compare data between two groups with normal distribution and homogeneity of variance (**B**,**C**,**E**); two-way ANOVA followed by Sidak’s multiple comparison test was used for time- and treatment-dependent analysis (**A**). Each bar represents the mean ± SD, the dots represent biological replication of the experiments. * *p* < 0.05, *** *p* < 0.001 vs. C, control. Scale bar: 100 µm.

To further investigate mitochondrial metabolism after OA treatment, we next exanimated ATP production from glycolysis (glycoATP) and from mitochondrial respiration (mitoATP). The results showed that the basal oxygen consumption rate (OCR) was higher after treatment with 500 µM OA (Figure 3A,B; *p* < 0.01). The total ATP production rate of HGrC1 cells exposed to OA (500 µM) was increased (Figure 3C; *p* < 0.001), which was mainly contributed by the mitochondrial respiratory pathway, as the glycoATP pathway showed little change (Figure 3D). Nile Red staining showed that the number of lipid droplets was decreased after treatment with OA at 500 µM for 24 h (Figure 3E,F; *p* < 0.05).

### 2.3. Glucose and Fatty Acid Transporter Expression

**Figure 4:**
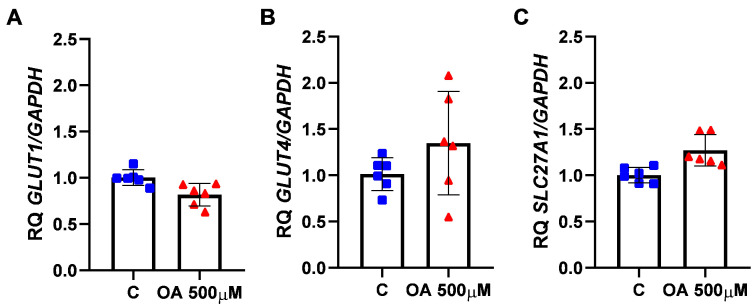
Glucose and fatty acid transporter gene expression after orotic acid (OA) treatment in human granulosa (HGrC1) cells. The effect of OA at 500 µM on (**A**) GLUT1, (**B**) GLUT4, and (**C**) SLC27A1 gene expression. The mRNA expression of the gene in vehicle-treated cells was set to 1.0. RQ, relative quantity. C, control (0.1% DMSO). A paired *t*-test was used to determine statistical significance. Data are expressed as mean ± SD, the dots represent biological replication of the experiments.

To determine whether changes in mitochondrial activity and ATP production correlate with substrate bioavailability, we next analysed glucose and fatty acid transporters after OA treatment. There were no statistically significant differences in glucose transporter (GLUT1, GLUT4) and fatty acid transporter (SLC27A1) gene expression (Figure 4A–C).

### 2.4. Effects of OA on Mouse Oocyte In Vitro Maturation

**Figure 5:**
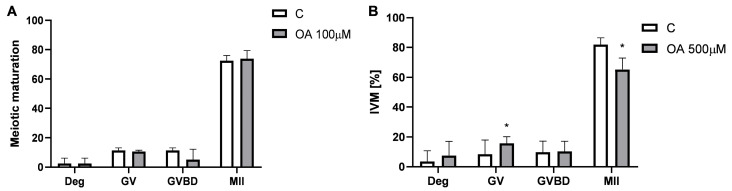
In vitro meiotic maturation of mouse oocytes after exposure to orotic acid (OA). The rate of metaphase II (MII), germinal vesicle breakdown (GVBD), germinal vesicle (GV), and degraded (Deg) oocytes in control (C) and OA at (**A**) 100 µM and (**B**) 500 µM groups. A paired *t*-test was used to determine statistical significance. Each bar represents the mean ± SD of three independent experiments. * *p* < 0.05 vs. C.

To access the effect of OA on oocytes, in vitro oocyte maturation was first examined. Of the oocytes that resumed meiosis, most underwent full meiotic maturation to the metaphase II (MII) stage in the control and 100 µM OA-treated groups (Figure 5A). However, the percentage of MII oocytes at the end of the culture period was lower in the 500 µM OA group than in the control group (82% vs. 64%) (Figure 5B; *p* < 0.05). We observed that oocytes treated with the high dose of OA were arrested or degraded at the GV stage (8% vs. 16%) (Figure 5B; *p* < 0.05).

### 2.5. Effect of Orotic Acid on Reactive Oxygen Species Production and Mitochondrial Activity on Mouse Oocyte That Matured In Vitro

**Figure 6:**
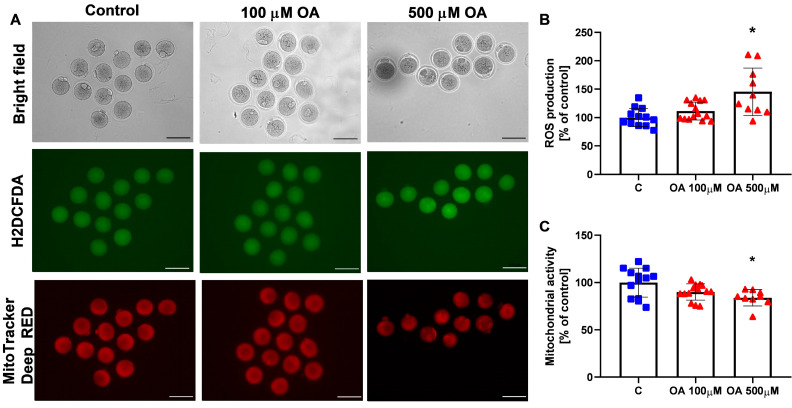
Effect of orotic acid (OA) on mitochondrial activity and ROS production. Effect of treatment of mouse oocytes with 100 µM and 500 µM OA on reactive oxygen species (ROS) production (**B**) and mitochondrial activity (**C**) after 18 h. Representative images of H2DCFDA labelling, indicating ROS concentration and MitoTracker Deep Red labelling, indicating mitochondrial activity (**A**). Control (C); orotic acid (100 µM and 500 µM). One-way ANOVA followed by Tukey’s multiple comparison test was used to determine statistical significance. Data are the mean ± SD of three independent experiments. * *p* < 0.05 vs. C, control. Scale bar: 100 µm.

After 18 h of culture, oocytes were labelled with Mitotracker Orange and H2DCFDA to assess their mitochondrial activity and intracellular reactive oxygen species (ROS) levels, respectively (Figure 6). There was a statistically significantly increase in the mean H2DCFDA fluorescence intensity between control oocytes and oocytes treated with 500 µM OA (Figure 6A,B; *p* < 0.05). The intensity of the Mitotracker Deep Red label (Figure 6A) was slightly but statistically significantly decreased in oocytes treated with 500 µM OA (Figure 6C; *p* < 0.05).

## 3. Discussion

Given how little research has been conducted on OA and the promises made in the public domain, we decided to evaluate its potential effect on ovarian function. The suggestion that dietary orotate might be beneficial to animals and humans was apparently based on its substantial natural levels in milk and dairy products [13]. A low level of OA in human milk contrasts with a remarkably high concentration in bovine milk (average 80 mg/L; ~500 µM), which exceeds the concentration of pyrimidine nucleosides and nucleotides. There are very few studies investigating the specialised functions of OA. This study is the first to investigate the effects of OA on energy homeostasis of healthy human ovarian cells and mouse in vitro oocyte maturation. We analyse OA mainly at levels of 100 µM and 500 µM (16–80 mg/L), which reflects the range of cow’s milk [3]. The enteral absorption for OA was only to 5–6% [3]. However, for consumers who consume several nutrients with orotate as a source, the total expected combined exposure would be more than 11 g/day [3]. Therefore, our doses represent pharmacological intervention and show that 100 µM OA has no effect on ovarian homeostasis, whereas 500 µM induces ATP production in granulosa cells (GCs) but reduces the efficiency of oocyte maturation in vitro.

GCs play an important role in maintaining ovarian function and are considered to be the most important cell type in the ovary besides the oocyte, influencing oocyte development and maturation. These cells interact with the oocyte to create a microenvironment for folliculogenesis and oocyte maturation. Specifically, GCs convert glucose to pyruvate, which is transported into the oocyte and enters the mitochondria to produce ATP [14]. In general, the key function of mitochondria is to produce energy through oxidative phosphorylation and lipid oxidation. In our previously published data, we observed that OA increased the mitochondrial membrane potential in HGrC1 cells [15]. We therefore ask whether this is associated with changes in the metabolic profile and ATP production. We observed that OA at a high dose (500 µM) stimulated the rate of oxygen consumption and ATP production while decreasing the content of lipid droplets (LDs) in granulosa cells. LDs are energy-storing organelles in cells that change dynamically in response to cellular energy needs [16]. Previous data have shown that rats fed a balanced diet supplemented with 1% OA have increased cardiac fatty acid oxidation and glycolysis, reflected in increased energy content, even in the pre-ischemic state [7]. Taken together, these results suggest that high doses of OA upregulate cellular bioenergetics. This induction is mediated by fatty acids. This is important because fatty acid metabolism in the granulosa cells surrounding the oocyte is important for maintaining metabolic homeostasis and may influence meiotic progression and survival of the enclosed oocytes [17].

Therefore, we next analysed the mitotic maturation of oocytes and found that OA at a dose of 100 µM did not disrupt mouse in vitro oocyte maturation, whereas OA at a dose of 500 µM inhibited this process by arresting oocytes at the GV stage. Reduced in vitro maturation was also correlated with a decrease in oocyte mitochondrial activity and an increase in ROS production. To our knowledge, this is the first study to describe the effect of OA on oocyte maturation in vitro. We found that high levels of OA disrupt ROS homeostasis by overproducing ROS. This can lead to a shift in the redox balance from eustress to distress, making oocytes susceptible to damage, particularly in the in vitro environment where protective maternal factors are absent. Homeostatic levels of ROS are necessary for normal oocyte maturation, fertilisation and embryo development [18]. Interestingly, OA has been detected in human follicular fluid (FF) at levels of 0.21 ± 0.27 µM [19]. This has important implications, as FF is the biological fluid that supports oocyte maturation, influences the effectiveness of oocyte fertilisation and affects embryo development of fertilised oocytes. FF is a mixture of granulosa and theca cell secretions and compounds diffusing from plasma [20]. Therefore, OA may directly affect the oocyte as a component of FF.

There are still several limitations to this study. Obtaining human oocytes for research is challenging due to ethical issues. Instead of human oocytes, we decided to analyse mouse oocytes, which has limitations that should be considered when interpreting research results. Mice and humans share a large part of their genome, but there are also significant differences in coding sequences and gene regulation. These differences can affect the results of studies when extrapolating from mouse models to humans. Another limitation of the experimental model used is that the effects of chronic treatment on OA are more difficult to determine. Therefore, the duration of treatment may have been too short to induce all effects in oocytes and granulosa cells.

## 4. Materials and Methods

### 4.1. Cell Culture of Ovarian Human Cell Lines

The human non-luteinised granulosa cell line HGrC1 was generously provided by Dr. Ikara Iwase (Nagoya University, Nagoya, Japan). HGrC1 cells originate from granulosa cells isolated from antral follicles measuring 3–5 mm [21]. They were cultured in phenol red-free DMEM (Sigma-Aldrich, St. Louis, MO, USA) supplemented with 2 mM L-glutamine and 10% fetal bovine serum (FBS). The human ovarian surface epithelial cell line HOSEpiC was purchased from ScienCell Research Laboratories (Carlsbad, CA, USA, Catalog No. 7310) and cultured in OEpiCM medium (Carlsbad, CA, USA). All cells were kept at 37 °C in a humidified incubator containing 95% air and 5% CO_2_. For experiments, cells were seeded into 96-well tissue culture plates (Nunc, Thermo Fisher Scientific Inc., Waltham, MA, USA) at a density of 8 × 10^3^ cells/well per well in 0.1 mL of completed medium and allowed to adhere for 12 h.

### 4.2. Quantification of Cell Viability

HOSEpiC and HGrC1 cells were exposed twice within 48 h to varying concentrations of OA (Sigma-Aldrich) (0.01, 0.1, 1, 10, 100, and 1000 µM) or to 0.1% DMSO as a control. Cell viability was assessed using the PrestoBlue™ HS Cell Viability Reagent (Invitrogen, Paisley, UK) following the manufacturer’s protocol. Briefly, PrestoBlue solution was added aseptically to each well at 10% of the total culture medium volume and incubated for 10 min. Background fluorescence was measured in control wells containing only culture medium or medium with OA, but without cells. The conversion of resazurin to resorufin was quantified by measuring fluorescence at 530 nm excitation and 590 nm emission using a Varioskan™ LUX multimode microplate reader (Thermo Fisher Scientific, Waltham, MA, USA). Data analysis was performed using SkanIt RE 6.1.1 software (ThermoFisher Scientific).

### 4.3. Determination of Lipid Droplet Accumulation

To evaluate lipid droplet accumulation in HGrC1 cells treated with either 500 µM OA or 0.1% DMSO (control) for 24 h, intracellular lipids were stained using Nile Red (Invitrogen, Paisley, UK). After two PBS washes, cells were incubated for 60 min with a 10 µM Nile Red solution prepared in serum-free medium. Following staining, cells were visualised with an Axiocam 503 bright-field fluorescence microscope (Zeiss, Jena, Germany; excitation at 590 nm). Fluorescence intensity was quantified using a Varioskan™ LUX multimode microplate reader (Thermo Fisher Scientific; excitation at 552 nm, emission at 636 nm), and data analysis was performed with SkanIt RE 6.1.1. software (ThermoFisher Scientific)

### 4.4. Analysis of Mitochondrial Function

A real-time ATP rate assay kit (Agilent, Santa Clara, CA, USA) was used to evaluate mitochondrial function following the manufacturer’s protocol with a Seahorse XFp analyzer (Agilent). HGrC1 cells were seeded in an 8-well XFp cell culture miniplate (Agilent, Santa Clara, CA, USA) and incubated overnight to allow attachment. Cells were then treated with OA at concentrations of 0.01 µM, 100 µM, and 500 µM, or with 0.1% DMSO as a control, for 24 h. Before starting the assay, the culture medium was replaced with Seahorse XF DMEM assay buffer (pH 7.4) supplemented with 2 mM glutamine, 1 mM pyruvate, and 10 mM glucose. Each well received 180 µL of this assay medium, and cells were incubated in a CO_2_-free incubator for 1 h. Measurements of extracellular acidification rate (ECAR) and oxygen consumption rate (OCR) were measured following sequential additions of OA, oligomycin (1.5 µM), and a combination of rotenone and antimycin A (Rot/AA, 0.5 µM). The use of oligomycin, an inhibitor of mitochondrial ATP synthase (complex V), together with Rot/AA, inhibitors of mitochondrial ETC electron transport chain complexes I and III, allowed real-time quantification of total ATP production and the specific contributions from glycolytic (glycoATP) and mitochondrial (mitoATP) pathways using the Seahorse analyzer. Data analysis was performed with Seahorse Wave software 1.0.0-749 (Seahorse Bioscience, Santa Clara, CA, USA) and exported to Prism 8 (GraphPad, La Jolla, CA, USA) for statistical evaluation. Protein quantification was conducted for data normalisation: cells were lysed with 30 µL RIPA buffer (ThermoFisher Scientific) containing protease inhibitors, centrifuged at 15,000 rpm for 15 min at 4 °C, and the supernatant protein concentration was measured using a Nanodrop spectrophotometer (DeNovix DS-11, DeNovix Inc., Wilmington, DE, USA).

### 4.5. RT-qPCR

Expression levels of the GLUT1 (*SLC2A1*, Hs00892681_m1), GLUT4 (*SLC2A4*, Hs.00168966_m1), and SLC27A1 (Hs01587911_m1) genes were assessed in HGrC1 cells following a 24 h treatment with either 500 µM OA or 0.1% DMSO (control) using real-time PCR. Total RNA was extracted and reverse transcribed utilising the TaqMan Gene Expression Cells-to-CT Kit (Applied Biosystems/Thermo Fisher Scientific, Waltham, MA, USA). Each assay included a negative control (all RT reaction components except the 20X RT enzyme mix) and a no-template control (all PCR components without cell lysate), prepared according to the manufacturer’s instructions. Gene expression was normalised to GAPDH (4310884E), and relative mRNA levels were calculated using the 2^−ΔΔCt^ method [22], with GAPDH serving as the reference gene. TaqMan Gene Expression Assays (Applied Biosystems/Thermo Fisher Scientific) were used as ready-to-use primers for all target genes.

### 4.6. Mouse Oocyte Collection

Oocytes were collected from mature female outbred OF1 mice (n = 12, aged 4–6 months) maintained in an animal facility under controlled temperature (22 ± 2 °C) and a 12 h light/dark cycle, with unrestricted access to food and water. Animals were sacrificed by cervical dislocation, and ovaries were excised. All procedures complied with national regulations for the care and use of laboratory animals and adhered to the European Union Council Directive 2010/63/EU of 22 September 2010. Reporting followed the ARRIVE guidelines [23].

Ovaries were transferred to drops of M2 culture medium and punctured with sharp tweezers to release oocytes from antral follicles. Cumulus cells, when present, were removed from oocytes by pipetting. Only oocytes at the germinal vesicle (GV) stage were selected for further analysis.

### 4.7. Assessment of Meiotic Maturation of Mouse Oocytes In Vitro

Groups of 9–14 GV oocytes were placed in drops of M2 medium containing either 0.1% DMSO (as a control) or OA at concentrations of 100 µM and 500 µM. These drops were covered with mineral oil in Petri dishes and incubated at 37 °C in an atmosphere of 5% CO_2_ to facilitate meiotic maturation. The cultures were observed after 2 h to monitor the resumption of meiosis and again after 18 h to assess the overall efficiency of meiotic maturation. Based on these observations, the oocytes were classified into three groups: GV oocytes, which had not resumed meiosis; GVBD oocytes, which had resumed meiosis and reached the germinal vesicle breakdown stage; and MII oocytes, which had extruded the first polar body and arrested at the second meiotic metaphase, indicating completion of meiotic maturation.

### 4.8. Assessment of Oocyte Mitochondrial Activity and Reactive Oxygen Species Levels

To evaluate mitochondrial function and reactive oxygen species (ROS) levels in oocytes after 18 h of culture with either 100 µM or 500 µM OA, or with 0.1% DMSO as a control, oocytes were stained with MitoTracker Deep Red (M22426; Invitrogen, Paisley, UK) for mitochondrial activity and with 2′,7′-dichlorofluorescein diacetate (H2DCFDA; D399; Invitrogen, Paisley, UK) for ROS detection. For dual staining, oocytes were incubated for 30 min at 37 °C in M2 medium containing 500 nM MitoTracker Deep Red and 10 µM H2DCFDA. After incubation, oocytes underwent two 5 min washes in M2 medium. Imaging was performed using a Zeiss Axiocam 503 bright-field/fluorescence microscope (Zeiss, Jena, Germany) and equipped with appropriate filters for each fluorophore. Fluorescence images were analysed with ImageJ software 1.52k (NIH, Bethesda, MD, USA). Each oocyte was manually outlined to calculate the mean fluorescence intensity. To determine background fluorescence, the intensity was measured in four areas adjacent to each oocyte, averaged, and subtracted from the oocyte’s mean signal. Results are expressed relative to the mean fluorescence intensity of control oocytes, which was set at 100 for each experiment.

### 4.9. Statistical Analysis

All experiments were performed in at least three independent experiments in independent triplicates. Normal distribution was analysed by the Shapiro–Wilk test and homogeneity of variance was tested by Levene’s test; the data were expressed as the mean ± SD. Student’s *t*-test was used for comparisons of data between two groups conforming to normal distribution and homogeneity of variance, one-way ANOVA followed by Tukey’s post hoc test was used for comparisons of data among multiple groups conforming to normal distribution and homogeneity of variance. Two-way ANOVA followed by Sidak’s multiple comparisons test was used for time and treatment dependant analysis. *p* < 0.05 indicated statistically significant differences (GraphPad Software 8.0.1, La Jolla, CA, USA).

## 5. Conclusions

Taken together, we observed that high-dose OA can affect the bioenergetic processes of ovarian cells, similar to previously published data. However, the lower dose of OA did not disrupt ovarian metabolism or oocyte maturation in vitro. It is important to note that during oral administration, the uptake of circulating OA by human erythrocytes and its return to the blood as uridine [24] can generally maintain orotate at low serum concentrations [25]. It is therefore safe to assume that normal dietary intake of orotate will not interfere with ovarian function. However, female reproduction may be adversely affected by extensive treatment for OA. As the risks of high-dose OA supplementation have significant clinical implications, further research is needed.

## Data Availability

The data presented in this study are available on request from the corresponding author.
